# Impairments of Biological Motion Perception in Congenital Prosopagnosia

**DOI:** 10.1371/journal.pone.0007414

**Published:** 2009-10-12

**Authors:** Joachim Lange, Marc de Lussanet, Simone Kuhlmann, Anja Zimmermann, Markus Lappe, Pienie Zwitserlood, Christian Dobel

**Affiliations:** 1 Institute of Clinical Neuroscience and Medical Psychology, Heinrich-Heine-University Düsseldorf, Düsseldorf, Germany; 2 Radboud University Nijmegen, Donders Institute for Brain, Cognition and Behaviour: Centre for Cognitive Neuroimaging, Nijmegen, The Netherlands; 3 Department of Psychology, University of Münster, Münster, Germany; 4 Institute for Biomagnetism and Biosignalanalysis, University of Münster, Münster, Germany; 5 Otto Creutzfeldt Center for Cognitive and Behavioral Neuroscience, Münster, Germany; University of Minnesota, United States of America

## Abstract

Prosopagnosia is a deficit in recognizing people from their faces. Acquired prosopagnosia results after brain damage, developmental or congenital prosopagnosia (CP) is not caused by brain lesion, but has presumably been present from early childhood onwards. Since other sensory, perceptual, and cognitive abilities are largely spared, CP is considered to be a stimulus-specific deficit, limited to face processing. Given that recent behavioral and imaging studies indicate a close relationship of face and biological-motion perception in healthy adults, we hypothesized that biological motion processing should be impaired in CP. Five individuals with CP and ten matched healthy controls were tested with diverse biological-motion stimuli and tasks. Four of the CP individuals showed severe deficits in biological-motion processing, while one performed within the lower range of the controls. A discriminant analysis classified all participants correctly with a very high probability for each participant. These findings demonstrate that in CP, impaired perception of faces can be accompanied by impaired biological-motion perception. We discuss implications for dedicated and shared mechanisms involved in the perception of faces and biological motion.

## Introduction

Face perception plays a crucial role in human social interaction. Humans use face information to assess the identity and emotional state of a person within fractions of a second. They can differentiate and recognize a vast number of individuals with seeming ease. Only a dysfunction of this socially important perceptual skill is a reminder of the remarkable capability of the human mind. A dysfunction of face perception, termed prosopagnosia [1], is characterized by a strong impairment to recognize familiar faces despite largely intact basic sensory and perceptual abilities. Prosopagnosia is a relatively rare deficit, mainly associated with acquired lesions of occipito-temporal regions, in which case it is termed ‘acquired prosopagnosia’ (AP) [1]–[4]. Recently, there is increasing evidence for dysfunctional face processing in individuals without any reported brain lesion or any other known neurological impairment. Such cases have been termed ‘developmental’ [e.g. 5], [6], stressing the early origin or, ‘congenital’, emphasizing the absence of pathological correlate [e.g. 7], [8]. Given that there are cases in the literature that are termed ‘developmental’ in the presence of a brain damage in early childhood, we use the term ‘congenital prosopagnosia’ (CP), even though it has not been proven so far that the symptoms are in fact present upon birth.

Acquired or congenital prosopagnosia has attracted much interest in the scientific community and a broader audience likewise. There is an ongoing debate as to whether the deficits are caused by stimulus-specific impairments related purely to faces, or by more general processing impairments, which affect multiple domains and apply to a broader range of stimuli.

The stimulus-specific view holds that cortical mechanisms are organized around the particular type of information processed, such as facial stimuli. In line with this view, imaging studies in neurologically intact subjects have identified a cortical area (fusiform face area, FFA) which responds specifically to faces but not to objects [9]–[13], but see [14]. In addition, cases of AP and CP seem to support the stimulus-specific view as they reveal (double) dissociations between face and object perception [1], [5], [15]. Another argument in favor of the stimulus-specific view concerns different perceptual mechanisms for the recognition of faces and objects. While objects are predominantly processed in a part-based fashion [16], [17], face processing seems to rely more on the holistic, or configural arrangement of its parts [18]–[20]. This is often demonstrated by turning images upside-down, which hampers configural perception. This so-called inversion-effect holds for faces [20], but is less prominent or even absent for objects that apparently are not configurally perceived [e.g. 12], [21].

The alternative view of domain-spanning, general mechanisms proposes that the mind is divided into functions associated with specific processes that apply to diverse domains and stimulus types. In this view, dissociations between faces and objects are explained by differences in the level of expertise [22] or in task demands [23], [24].

While some studies on prosopagnosia found little or no evidence for an impairment of visual recognition of stimulus categories other than faces [25], [26], recent findings argue in favor of prosopagnosia as a more general impairment of configural or holistic processing. For example, subjects with CP were impaired in processing artificial stimuli whose global form (e.g. a letter) is composed of smaller, local elements (often from the same category, such as letters) [7]. Moreover, individuals with CP showed abnormal components of event-related potentials in response to static human bodies [6]. The degree of the impairment, however, depended on the specific case, the extent of prosopagnosia, and/or the tasks [5], [6], [26], [27]. A recent fMRI study found normal BOLD responses to faces in the FFA of a prosopagnosic individual, arguing for dysfunctions within a complex network for face processing [28].

Proponents of the stimulus-specific view argue that general mechanisms are not necessary as long as cortical mechanisms specialized for face processing are also able to process other types of stimuli, by analyzing features that these stimuli have in common with faces (for a discussion see [9]). Thus, a real challenge for claims about a stimulus-specific or a general impairment is to show a deficit in prosopagnosic individuals for stimuli that do not share any common features with faces, but that share their processing mechanisms with face stimuli.

To meet this challenge, we consider face and biological-motion stimuli ideally suited. The term ‘biological motion’ describes the movement of human (or animal) bodies or its parts. It involves hand, eye, lip, or whole-body movements, which, together with faces, constitute crucial ingredients of social cognition and interaction [29]. Face processing is usually tested with static images that are rich in visual information. In contrast, perception of whole-body biological motion is often assessed with dynamic displays of a few point-lights, providing only sparse visual information [30], [31].

Lip movements constitute stimuli that share characteristics with biological-motion and face processing. The lips are an important part of the face, as humans can extract rich visual information from lip movements in the absence of any other facial information. Lip movements are dynamic, with constant form changes that are essential for speech-reading, for example. Thus, lip movements link face and dynamic biological-motion perception.

Although they are very different with respect to their visual features, face, lip, and whole-body movements have a lot in common. First, they all show a strong inversion effect [20], [21], [32], [33], which is evidence for their reliance on configural processing [20], [21], [34], [35]. Also, all stimuli are relevant from early childhood on [36]. In addition, the perception of lip movements, whole-body movements and faces involves common cortical networks, as integrated parts of the social-cognition network [29].

The objective of the present study is to determine whether the perceptual deficits found in CP are restricted to the recognition of faces, or also to recognition of biological motion and lip movements. If CP is caused by an impairment restricted to face perception, impairment of this process is unlikely to affect the processing of other stimulus types such as biological motion. On the other hand, if CP arises from more general deficits, prosopagnosic individuals might have problems with faces, lips, and body motion. To investigate these hypotheses, we tested five individuals suffering from CP that had participated in earlier studies on face perception, as well as ten matched controls. We used different kinds of stimuli on biological motion (in upright and inverted orientations), such as silent lip-movements, and point-light displays of human whole-body movements.

## Results

In the following, we report results for congenital and control participants from different tasks on silent lip-reading and point-light biological motion. We describe separately the recognition rates and response latencies for all tests both by group statistics and at a single-case level.

### Lip Reading

**Figure 1:**
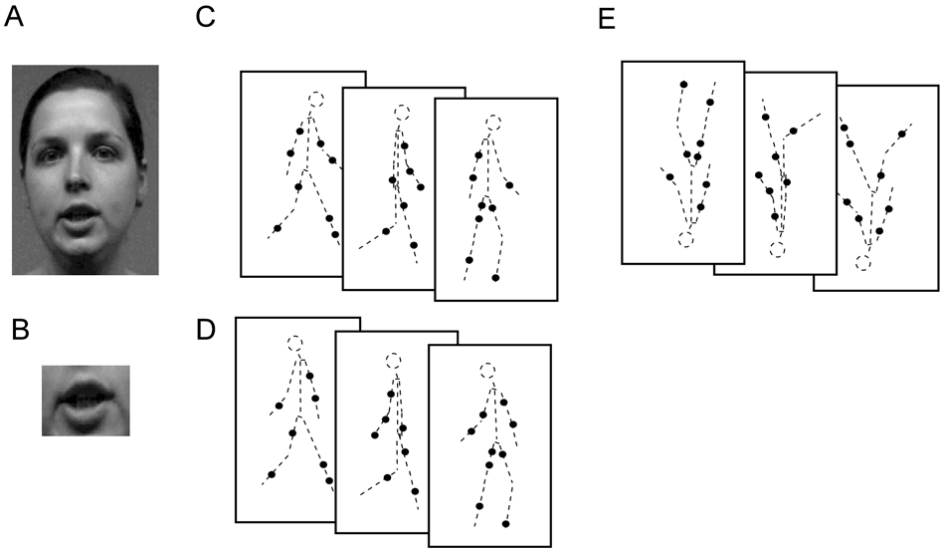
Illustration of the stimuli. A, B) single frames of the: whole-face (A) and mouth-only (B) lip-reading movies (speaking “9” in German). C, D) Illustration of the experiments on point-light biological motion: C) Three single frames of a normal walking sequence. The sequence illustrates an SFL-walker, i.e. the single dots randomly change their position on the stimulus limbs each frame (see Experimental Procedure for details). The frames could face either to the left or to the right, played forwards (Left/Right-Discrimination Task) or they could be played either forwards or in reversed order (Forward/Backward-Discrimination Task). D) Illustration of the Coherent/Incoherent-Discrimination Task. Upper and lower parts of the stimulus could be either moving in the same direction (coherent, as illustrated in C) or they could be flipped by 180° (incoherent). E) Illustration of an inverted ‘SFL-Walker’. The dashed lines are only for demonstration and not shown in the real stimulus.

To test lip-reading performance, subjects viewed silent videos of actors speaking number words (1 to 10), which they had to recognize (Fig. 1A, B; Movie S1).

### Recognition Rates

**Figure 2:**
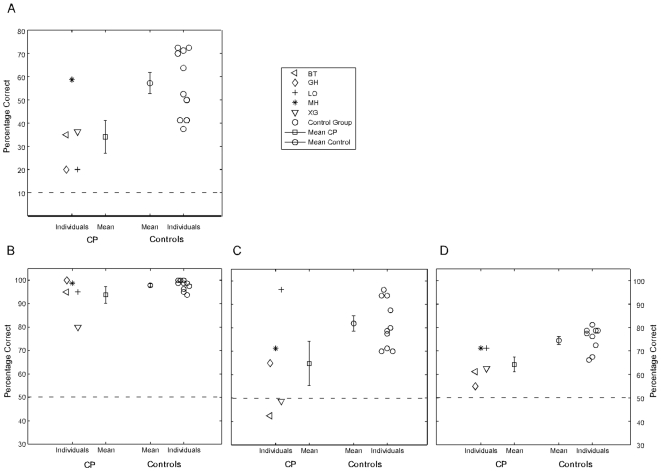
Individual accuracy rates and the group average for CP and Control group for the Discrimination tasks. Percentage correct is shown for the Lip reading task (A), Left/Right Task (B), Coherent/Incoherent Task (C), and Forward/Backward Task (D). Dashed lines indicate chance level. The legend applies to all plots. Group results are presented as mean ± 1 SEM.

CP participants made significantly more errors than control participants (F(1,13) = 8.21, p = .01; Figure 2A). Error rates were higher for inverted than for upright stimuli, averaged over groups (accuracy: F(1,13) = 9.95, p<.01). The interaction of orientation and face information (mouth-only or whole-face) was significant (F(1,13) = 5.76, p<.05). Post-hoc analysis revealed that the inversion effect was stronger for inverted whole-faces than for inverted mouth-only stimuli (whole-faces: t(14) = −3.18, p<.01; mouth-only: t(14) = −1.99, p = .07). No significant interactions were found for group x orientation (F(13) = .56, ns.), for group x face information (F(13) = .4, ns) nor for group x orientation x face (F(13) = 1.8, ns).

### Response Latencies

No significant differences were found for response latencies between groups (F(1,13) = 0.19, ns). One control participant showed extremely long latencies of 6.38 s, which is more than 2.5 standard deviations above the control group's mean (Mean: 3.08 s, SD: 1.21 s; see Figure S1A). After exclusion of these data from the analysis on latencies, there was a trend (F(1,12) = 4.23, p = .06).

Response latencies for inverted and for upright stimuli did not differ significantly (F(1,13) = 0.11, ns). The interaction of group x orientation x face information was significant (F(13) = 6.2, p<.05). Post-hoc analysis for the control group revealed no inversion effect for faces (F(9) = .43, ns), but for mouths (t(9) = 2.4, p<.05). No significant inversion effect was found for the CP group (mouths: t(4) = −.9, ns; faces: t(4) = −.3, ns. No significant interactions were found for orientation x face information (F(1,13) = 0.20, ns), group x orientation (F(13) = 2.6, ns) nor for group x face information (F(13) = 1.2, ns).

In summary, the group of CP individuals revealed impaired perceptual skills for lip-reading numbers, evidenced by accuracy and/or latency data. Inspecting the data on a single case basis revealed that only MH had no difficulties with this task.

### Recognition of Point-light walker (PLW)

**In the Left/Right Discrimination Task**, participants decided whether point-light walkers faced to the left or to the right.

### Recognition rates

Performance did not differ between CP subjects and controls (F(1,13) = 2.46, p = .14, ns; Figure 2B). Participants responded more accurately (F(1,13) = 8.02, p<.01) to upright stimuli. Also, responses to the classic walker were more accurate (F(13) = 13.0, p<.01). None of the interactions were significant: group x orientation (F(13) = 1.6, ns), group x stimulus type (F(13) = 2.5, ns), group x orientation x stimulus type (F(13) = 0.4, ns).

Evaluation at the individual subject level revealed that one prosopagnosic subject (XG) performed relatively poorly, with only 80 percent correct responses, which is more than two standard deviations below the group average of controls. The recognition rates of two prosopagnosic subjects (BT and LO) were still one standard deviation below of the control group's mean (Figure 2B).

### Response Latencies

Performance did not differ between CP subjects and controls (F(1,13) = 1.11, ns; Figure S1B in Supporting information). Participants responded faster (F(1,13) = 44.68, p<.001) to upright stimuli. Also, responses to the classic walker were faster (F(13) = 22.2, p<.01). None of the interactions were significant (group x orientation (F(13) = .05, ns), group x stimulus type (F(13) = 0.88, ns), group x orientation x stimulus type (F(13) = 3.1, ns)).

Analysis at an individual level for latencies revealed that XG's reaction times were more than three standard deviations above the control group's mean (XG: 2.88 s; controls: 1.77±.35 s; Figure S1B in Supporting Information). The latencies of the remaining four CP participants were within the normal range (1.78 s on average).

Overall, the individual analyses suggested that there was a difference between control subjects and three individuals with CP. The lack of statistically significant differences on the group level may thus be due to the ceiling effect. The following Coherence/Incoherence and Forward/Backward tasks are more difficult [37]. We thus expected more pronounced group differences with these tasks.

**In the Coherence/Incoherence Discrimination Task**, point-light walkers were presented with the upper and lower body halves facing and moving in opposite directions (incoherent, Fig. 1D; Movie S6, S7 in Supporting Information) or with normal body-part orientation (coherent, Fig. 1C; Movie S2, S3 in Supporting Information). Subjects had to decide on the coherence of upper and lower body.

### Recognition rates

CP subjects made significantly more errors than control subjects (F(1,13) = 4.65, p<0.05; Figure 2C). Overall, responses were more accurate (F(1,13) = 8.50, p<.01) to upright than to inverted stimuli. There was no significant effect for walker type (F(1,13) = .06, ns). No significant interactions were found: group x orientation (F(1,13) = 1.45, ns), group x stimulus type (F(1,13) = 0.20, ns), group x orientation x stimulus type (F(1,13) = 1.19, ns).

Analysis at the single-subject level revealed that two CP participants performed at chance level (BT: 42.5%, XG: 49% correct; Figure 2C). Recognition rates for GH (65%) and MH (71%) were not better than the performance of the worst subjects from the control group. Only LO's performance (96%) was within the range of the control group.

### Response Latencies

There was no effect on latency (F(1,13) = 0.27, ns; Figure S1C Supporting Information). Overall, responses were slightly faster (F(1,13) = 4.40, p = .06) to upright than to inverted stimuli. Also, subjects responded faster (F(1,13) = 10.60; p<.01) to the classic walker. No significant interactions were found: group x orientation (F(1,13) = 1.50, ns), group x stimulus type (F(1,13) = .12, ns), group x orientation x stimulus type (F(1,13) = .05, ns).

Overall, four of five individuals of the CP group showed a considerable impairment in this task, evidenced by lower recognition rates.

**In the Forward/Backward Discrimination Task**, subjects had to decide whether point-light walkers were moving forward or backward.

### Recognition rates

The number of errors was significantly higher in the CP than in the control group (F(1,13) = 9.53, p<0.01; Figure 2D). Overall, responses to upright walkers were more accurate (F(1,13) = 19.86; p<.001) than to inverted stimuli. Also, subjects responded more accurately (F(1,13) = 21.54, p<.01) to the classic walker. No significant interactions were found for the interaction group x orientation (F(1,13) = 0.03, ns), group x stimulus type (F(1,13) = 2.14, ns), nor group x orientation x stimulus type (F(1,13) = 0.13, ns).

At the individual level, three CP subjects (BT, GH, and XG) performed worse than any control subject (Figure 2D). Only LO and MH of the CP group performed approximately within range of the controls, with three of the ten controls performing slightly worse.

### Response Latencies

There were no significant differences for response latency (F(1,13) = .07, ns; Figure S1D in Supporting Information). Overall, responses to upright walkers were faster (F(1,13) = 7.96, p = .01) than to inverted stimuli. Also, subjects responded faster (F(1,13) = 37.23; p<.01) to the classic walker. No significant interactions were found for the interaction group x orientation (F(1,13) = .43, ns), group x stimulus type (F(1,13) = 0.56, ns), nor group x orientation x stimulus type (F(1,13) = 0.08, ns).

At the individual level, latencies of all CP subjects were within normal range (Figure S1D in Supporting Information).

In all, this task also revealed poor performance of the CP group. Three individuals with CP performed worse than any of the controls, the remaining two within the lower range of the control group.

We performed additionally a stepwise discriminant analysis, to examine the probability of group membership for each participant. Of all of the analyzed variables from the above four experiments, three variables went into the model (entry criterion: F = 3.84; remove criterion: F: 2.71) in the following order: correct responses in lip reading of inverted whole faces, correct responses to upright coherent/incoherent walkers, and latency to respond to upright SFL forward/backward walkers. The summarized statistics for individual cases demonstrated that all participants (i.e. 100%) were classified correctly. The probability of belonging to the predicted group given the discriminant score was 100% for individuals with CP, except for MH whose probability was 98.3%. Similarly, with the exception of two subjects (92% and 98%), all control participants reached a probability of 100% to belong to their group. The same results were reached with a leave-one-out cross-validation analysis (e.g. MHs probability to belong to the group of CPs was 97.6%).

## Discussion

Prosopagnosia is most prominently characterized by a deficit in recognizing familiar faces. It has been intensely debated whether this impairment is stimulus-specific to faces or whether it also affects other stimulus categories. As reviewed in the introduction, the evidence for both positions is mixed. The objective of the present study was to address this question and the general nature of prosopagnosia, by testing congenital prosopagnosics (CP) on a number of biological-motion stimuli. Even though faces and biological-motion stimuli have quite different visual properties, they show an interesting overlap in terms of perceptual results [21], [32], [33], underlying cortical networks [6], [29], and putative processing mechanisms [20], [34], [35].

We assessed the perception of biological-motion stimuli in five prosopagnosic individuals who had previously been diagnosed as severely impaired in recognizing famous faces, but who showed normal performance in object perception [8], [38]. We attested deficits in prosopagnosics with stimuli that differ from faces in terms of visible form, geometric features, and/or format. Deficits were predicted on the basis of earlier findings that were interpreted as evidence for a common neural mechanism underlying face and biological-motion processing. We showed that participants with CP were considerably impaired in silent lip-reading and in the perception of whole-body motion, as indexed by increased error rates and/or prolonged latencies. This result was evident at the group level, but also on a single-case basis. Therefore, the results as such demonstrate that the face-specific dysfunction in CP can be accompanied by impaired perception of other stimulus types.

This finding raises the important question as to (1) whether face and biological-motion perception can be impaired independently from each other, (2) whether both rely on a common mechanism, implying a mandatory association of symptoms, or (3) whether both common and separate processes are involved. If biological motion and face perception rely on one and the same mechanism, we should find an association of symptoms in each case. A single report of a dissociation of symptoms would be an argument against an exclusive, common, domain-general mechanism. As such, participant MH from our prosopagnosic group might be a case in point. While four of the five prosopagnosic individuals were impaired on recognition rates and/or response latencies in all tasks, MH was clearly impaired in the Coherent/Incoherent Discrimination task, but his performance in the other tasks was within the normal or lower range of the control group. This single case thus seems to provide evidence for separate processes of face, lip, and body perception, which can be selectively impaired. Note, however, that the dissociation is certainly not complete: MH often performed in the lower range of the controls' performance, and discriminant analysis assigned him to the CP group.

The idea that independent processes are involved for faces and human bodies is supported by neuroimaging studies reporting spatially non-overlapping areas for processing faces and human bodies in higher visual areas [39]–[43]. Also, Duchaine et al. demonstrated a dissociation between impaired face processing and normal body processing in a prosopagnosic individual [44]. Note that Duchaine et al. used different stimuli and tasks than we did (headless full-body displays of static human bodies in a delayed matching to sample task). Differences between our study and Duchaine et al. might therefore be explained by different stimuli and/or tasks. For example, full bodies can be recognized more easily on the basis of single body parts, facilitating the task for prosopagnosics. It would be interesting to test our prosopagnosic individuals on full-body stimuli in future studies.

There is also evidence against the strict view of independent impairments for face and body perception. A recent study indicates that the neural substrates involved in body and face perception are less categorically segregated in prosopagnosic individuals than in normal subjects [45]. Another study has revealed anomalous ERP components in response to both face and whole-body stimuli in prosopagnosics [6]. It has also been shown that the deficits in prosopagnosia are rather inhomogeneous, showing a considerable variability in recognition of faces but also in the extent of putatively associated deficits [6], [7], [45].

The third possibility mentioned above is that the two views, independent vs. common impairment of body and face processing, are not mutually exclusive. Although human faces and bodies, and in particular their respective parts, might be processed initially in separate brain areas by domain-specific mechanisms, general mechanisms common to all biological stimuli might also exist. In line with this hypothesis, recent studies argued that impaired face perception might reflect a specific symptom of a general impairment in prosopagnosia [6], [46], [47]. These data, together with our own, argue that over and above dedicated processes for face and biological-motion perception, a common mechanism is involved, which can be impaired in CP. Note that such a view does not predict a compulsory dissociation, and is compatible with the bulk of the available data.

What could constitute the underlying common characteristics of such a domain-general impairment of face, lip movement, and human body perception? It has been proposed that the processes involved in the perception of faces, human bodies and their movements, as well as of lips, are all configural in nature [19], [20], [33]–[35]. In line with this, we found faster responses and/or lower error rates for all stimulus types when presented in upright as compared to inverted orientation. Thus, all of our stimuli induced a strong inversion-effect. It has been shown that configurally perceived stimuli, such as faces, induce a stronger inversion effect than non-configurally perceived objects [19], [21], [32], [33]. Our results support the view of configural perception of bodies and lips [19], [20], [33]–[35]. The hypothesis of impaired configural perception in prosopagnosia has been tested before, however, with mixed results [2], [3], [5], [26], [46], [48]–[52]. Note, however, that under the third hypothesis, there are dedicated and general mechanisms responsible for face processing, and these can be individually (or jointly) impaired.

In line with such a dysfunction of configural processing, our prosopagnosic individuals showed severe deficits in the silent lip-reading of mouth displays presented upright. Furthermore, CPs were impaired in all discrimination tasks with upright point-light walkers. However, the importance of configural information differed between stimuli and tasks. While direction (left/right) discrimination tasks can be solved either by employing single dots as local cues [53], or by the configural constitution of the body [37], [54], forward and backward movements can only be discriminated on the basis of configural processing [37], [54]. Consequently, the inversion effect was weakest for left/right discrimination, and strongest for forward/backward discrimination. Furthermore, differences between CP and control groups correlated with the amount of configural stimulus information. Differences were largest for the forward/backward task and smallest for the left/right task. Even though overall performance in the left/right discrimination task was similar between groups, the individual data strongly suggest that at least three participants with CP do show deficits also in this task. Thus, while most participants reached ceiling, those who did not were participants of the CP group.

Several studies reported an reduced or absent inversion effect for configurally perceived stimuli in prosopagnosia, or even an inversion-specific superiority of prosopagnosic individuals [2], [7], [48], [49]. In the present study, we found an inversion-specific superiority-effect of CPs for lip-reading: While the control group revealed an inversion effect for the mouth-only condition, this inversion effect was absent for CPs. Similar results for faces have been explained by a feature-based strategy of prosopagnosics, to overcome their deficits in configural processing [2], [48], [49]. However, we did not observe an inversion-specific superiority-effect in CP for whole-body movements. One explanation is that point-light biological-motion stimuli do not contain entities that can each be recognized when presented in isolation, as is the case for face parts. Whereas eyes, mouth, nose, hairline, etc. each have a very specific form, which could be recognized by prosopagnosic individuals even if stimuli are inverted, the “limbs” in a point-light body are all quite similar and provide most useful information if recognized configurally. A feature-based strategy would thus not be helpful for the recognition of inverted point-light displays of human movements.

The role of configural processing as an underlying common mechanism for the development of face and body perception was emphasized in a recent review [55]. Support comes from individuals suffering from developmental disorders, such as autism or Asperger syndrome, who are impaired in the recognition of faces [56]–[58], and of point-light displays of biological motion [59]. Given the high prevalence of CP in families, we regard it as one of the challenging, but promising approaches for future studies to identify children that suffer from face perception impairments, and to investigate how face and biological-motion perception develop.

Regardless of the specific neural mechanisms, neural structures shared by face and biological-motion perception may be impaired in CP. It has been suggested that the perception of faces, lips and whole-body movements is subserved by distributed but partially shared anatomical and functional networks [21], [29]. An impairment in shared parts might therefore account for our results. A candidate cortical region is the FFA, which plays a crucial role in face perception [9], [12] and is typically damaged in acquired prosopagnosia [1], [3]. Several studies have linked the FFA and adjacent areas to the perception of human bodies [41], [60], [61]. But note that imaging studies on prosopagnosic individuals revealed intact BOLD activity in FFA despite impaired face perception [28]. The superior temporal sulcus (STS) has also often been associated with face and biological-motion perception [29], [62], [63]. Hence, these areas form candidate cortical regions for an impairment of a common cortical network. In agreement with this hypothesis, a recent study revealed anomalous ERPs when individuals with developmental prosopagnosia viewed static images of human bodies or faces, compared to objects [6].

In sum, in support of and strengthening recent findings, our results demonstrate that the face-specific impairment in CP can be associated with a deficit in lip and body perception. One subject (MH) was impaired in Coherent/Incoherent Discrimination tasks but less so in the other tasks, which points to a partial dissociation between face and biological-motion processing. This finding, together with other data from the literature, argues against the view that faces and biological motion are exclusively subserved by one and the same mechanism. We argued in favor of a mixture of dedicated and shared processes for faces and biological motion, and proposed configural processing as a good candidate for the shared mechanism. Future studies combining neuropsychological and neurophysiological methods may shed more light on the common neuronal structures that are crucial for the processing of these very different stimuli.

## Materials and Methods

### Subjects

Fifteen subjects (5 congenital prosopagnosics (CP) and 10 controls) contributed data to the study. All participants had normal or corrected-to-normal vision and signed an informed consent, stating that the aims of this study had been clarified to them, and gave their agreement to a potential publication of the data in anonymous form. All subjects (except one control subject) were naïve regarding the stimuli and the aim of the study.

All participants gave their written consent to participate in the study. The study falls under the ethical approval of the “Kommission der Ärztekammer Westfalen-Lippe und der Medizinischen Fakultät der Westfälischen-Wilhelms Universität Münster”.

#### Congenital Prosopagnosia Group

Five individuals (three females, mean age 39±17.6 years) suffering from CP participated in the study (see Table 1 for details on age, gender, education, and profession).

**Table 1:** Characteristics of congenital participants (CP) and the matched control group.

Participant		Age	Gender	Years of school	Profession	Matching controls
*CP*						
	LO	22	Female	13	Student	MX, KS
	BT	27	Female	12	Employee	AP, BX
	GH	59	Female	13	Self-employed	BT, PZ
	MH	30	Male	13	Software engineer	CZ, AN
	XG	57	Male	13	Professor	DP, BW
*Controls*						
	MX	20	Female	13	Student	
	KS	24	Female	13	Student	
	AP	27	Female	13	Doctor	
	BX	27	Female	13	Student	
	BT	58	Female	12	Med-tech. assistant	
	PZ	57	Female	12	Professor	
	CZ	28	Male	13	Student	
	AN	29	Male	14	Director	
	DP	57	Male	14	Engineering technician	
	BW	59	Male	12	Engineering technician	

Three members of the CP group (GH, MH, and XG) have been described in detail elsewhere [8], for LO and BT, see [38]. We briefly describe the testing of basic visual functions and discriminative face processing skills. Summarized results can be found in Table S1 (Supporting information); details on the tasks can be found in (8, 38).

All five participants displayed normal performance on a variety of object-perception tests (including the Visual Object and Space Perception battery (VOSP) [64]). BT performed below the critical cutoff in two subtests of the VOSP (progressive silhouettes, position discrimination). LO performed at cutoff level in the screening-test and progressive silhouettes of the VOSP.

All five participants of the CP group were strongly impaired in recognizing famous persons from face that they knew by name (Bielefelder Famous Faces Test [65]). Prosopagnosic subjects responded much slower to faces than to eyeglasses in a delayed-matching-to-sample task (for a full description of all tests, see [8]). All prosopagnosic subjects also participated in a study in which faces, houses, or sugarbowls had to be recognized based on configural (blurred images) or featural (scrambled images) information. All subjects with CP were strongly impaired for faces in general, but the strongest effects, with no overlap at all between prosopagnosics and controls, were visible in the blurred-faces condition [38]. Finally, in an behavioral experiment, all five participants recognized less famous faces than controls, and displayed a reduced or even absent face-inversion effect [66].

Apart from CP, these subjects suffered from no other known perceptual or neurological impairments. The participants received a reimbursement of 30 €.

#### Control Group

For each CP individual, we selected and matched two unimpaired participants with respect to age, gender, and educational level/profession. These 10 subjects formed the control group (see Table 1 for details). The mean age of the control group was 38.6±16.7 years. All control subjects had been known to at least one of the authors for several years; none reported any perceptual or neurological impairment or difficulty in recognizing people or faces.

### Stimuli and Tasks

Two main stimulus categories were used: lip-reading stimuli and point-light displays of human movements. All stimuli were presented on a laptop (Apple iBook G4) with display size of 24.5×18.4 cm and resolution of 1024×768 pixels. Viewing distance was approximately 80 cm. Tasks were presented in separate blocks, administered in a single session, with short breaks between blocks.

#### Lip Reading

Video sequences of three females speaking German number words (1 to 10) were recorded using a digital camera (Canon, MV500i, Canon Inc., Japan). Two sequences were used as test stimuli; the third was used as a warm-up before each experiment. Recordings were taken of the face in frontal view, with a grey background. The video sequences were cut into short movies (2s on average) showing the articulation of a number word. Recordings were edited in two ways: for the mouth-only movies, a rectangle comprising only the mouth was cut out (Figure 1B), for the other movies, the whole face was used (Movie S1). Movies presenting the whole face were in 10×7 cm format, mouth-only movies were 2.5×2 cm in size. All stimuli were presented upright and inverted, resulting in 80 trials (10 numbers x 2 conditions (mouth/face) x 2 orientations (upright/inverted) x 2 actors).

Stimuli were presented in random order. Participants were instructed to press the spacebar as soon as they recognized the number, which they subsequently specified by means of the designated key on the keyboard, followed by the “return” button. The time between the onset of the stimulus and the spacebar press was recorded as the latency. The next movie started 500 ms after hitting the “return”-button. Recognition rates and latencies were assessed. To familiarize subjects with the video presentations, instructions, and response modalities, a test trial preceded the actual experiment.

#### Point-light stimuli

We conducted three common tests for whole-body biological-motion perception: a Left/Right Discrimination Task, a, Coherent/Incoherent Discrimination Task, and a Forward/Backward Discrimination Task (described below in more detail. Two computer-generated stimulus types were used within each test: a “classic walker” [67] and a “single frame-lifetime (SFL) walker” [68].

The classic walker consisted of twelve light points representing ankles, knees, hips, wrists, elbows and shoulders (Movie S2). The SFL walker consisted of eight light points located at random positions on the four limbs (arms and legs) (Movie S3). Each point was flashed at random positions on the four limbs in each animation frame. A single frame lasted 16 ms. Compared to classic walking, the SFL walker strongly reduces the local motion information, as well as the possibility to use local cues from single light points for recognition [68].

Both walker types appeared as if walking on a treadmill. Stimulus size was always 4×2 cm. Size of the light points was five pixels, and walking speed was one cycle per 1.4 s. The stimuli were centered at a randomly selected location within 2.5 cm horizontally and 1 cm vertically from the centre of the screen.

#### Left/Right Discrimination Task

Half of the stimuli was facing and walking to the left, the other half was facing and walking to the right. In addition, stimuli were inverted (Movies S4, S5), resulting in a total of 80 trials (10 repetitions x 2 facing directions (left/right)*×*2 walker types (classic/SFL)*×*2 orientations (upright/inverted)), presented in random order. Stimuli remained visible until a response button was pressed. The next stimulus appeared 200 ms after the response. Error rates and reaction times were recorded. Sixteen practice trials preceded the actual task in order to familiarize subjects with the instructions, stimuli, and response modalities.

#### Coherent/Incoherent Discrimination Task

For half of the stimuli, upper and lower body of the walker were oriented in the same direction (coherent, Movie S2, S3), for the other half, upper and lower body parts were in opposite directions (incoherent, Movie S6, S7) [37], [69]. Stimuli were also presented inverted, resulting in a total of 80 trials (10 repetitions x 2 conditions (coherent/incoherent) x 2 walker types (classic/SFL) x 2 orientations (upright/inverted)).

Subjects were informed that a forward walking PLW would be presented. Their task was to judge whether upper and lower body were oriented in the same or opposite direction.

Subjects were instructed to give their answers by pressing keys on the keyboard. Sixteen practice trials preceded the test block.

#### Backward/Forward Discrimination Task

Half of the stimuli were walking forward, the other half backward [37]. Walkers were also inverted, resulting in 80 trials (10 repetitions x 2 walking directions (backward/forward) x 2 walker types (classic/SFL) x 2 orientations (upright/inverted)).

Subjects indicated via button press whether the stimulus was moving forward or backward (upward arrow key for forward, downward arrow key for backward). Walkers could either be oriented to the left or to the right, which was irrelevant for this task. Sixteen practice trials preceded the actual experiment.

## Supporting Information

Figure S1 Individual reaction times and the group average for CP and Control group for the Discrimination tasks. Response latencies are shown for the Lip-reading task (A), Left/Right Task (B), Coherent/Incoherent Task (C), and Forward/Backward Task (D). The legend applies to all plots. Group results are presented as mean±1 SEM.
(0.41 MB TIF)

Table S1 Test scores and results from neuropsychological test batteries and other experiments for prosopagnosic participants and matched controls.
(0.08 MB DOC)

Movie S1 Example movie of the stimuli for lip-reading. Example of a whole-face presentation in the number-recognition experiment (speaking “9” in German).
(2.22 MB MOV)

Movie S2 Example movies of the stimuli on point-light biological motion. ‘Classical walker’-stimulus moving forwards, facing to the right.
(0.18 MB MOV)

Movie S3 Example movies of the stimuli on point-light biological motion. ‘SFL walker’-stimulus moving forwards, facing to the right.
(0.17 MB MOV)

Movie S4 Example movies of the stimuli on point-light biological motion. Inverted ‘classical walker’-stimulus (see Movie S2).
(0.18 MB MOV)

Movie S5 Example movies of the stimuli on point-light biological motion. Inverted ‘SFL walker’-stimulus (see Movie S3).
(0.17 MB MOV)

Movie S6 Example movies of the stimuli on point-light biological motion. Incoherent ‘SFL walker’-stimulus.
(0.18 MB MOV)

Movie S7 Example movies of the stimuli on point-light biological motion. Incoherent ‘classical walker’-stimulus.
(0.17 MB MOV)
