# Supplementary material for: Impairments of Biological Motion Perception in Congenital Prosopagnosia
Source: PLoS One. 2009 Oct 12;4(10):e7414. doi: 10.1371/journal.pone.0007414 (PMC2756626; doi:10.1371/journal.pone.0007414)
Supplement: Table S1 — Test scores and results from neuropsychological test batteries and other experiments for prosopagnosic participants and matched controls. (0.08 MB DOC) [file pone.0007414.s002.doc]

Table S 1:

Test scores and results from neuropsychological test batteries and other experiments for prosopagnosic participants and matched controls

| Task | | Controls | GH | MH | XG | LO | BT |
| --- | --- | --- | --- | --- | --- | --- | --- |
| **Visual Object and Space Perception Battery** | | | | | | | |
|  | Screening | 20 | 18 | 20 | 19 | 15 | 18 |
|  | Incomplete Letters | 20 | 20 | 20 | 20 | 20 | 20 |
|  | Silhouettes | 26 | 27 | 29 | 22 | 16 | 16 |
|  | Object Decision | 18 | 20 | 18 | 18 | 18 | 18 |
|  | Progressive Silhouettes | 8 | * | 4 | 10 | 9 | 13 |
|  | Dot count | 10 | 10 | 10 | 10 | 10 | 10 |
|  | Position Discrimination | 20 | 19 | 20 | 20 | 20 | 16 |
|  | Number Location | 10 | 10 | 9 | 10 | 10 | 10 |
|  | Cube Analysis | 10 | 10 | 10 | 10 | 10 | 10 |
|  | Snodgrass Picture Naming | 100 | 97 | 100 | 97 | 100 | 100 |
|  | |  |  |  |  |  |  |
| **Face Perception** | | | | | | | |
|  | Bielefelder Famous Faces Test (% recognized faces from visual cue) | 73 | 30 | 31 | 47 | 3 | 40 |
|  | Benton Facial Recognition Test (recognized items) | 48 | 48 | 48 | 43 | 49 | 39 |
|  | |  |  |  |  |  |  |
| **Delayed Matching to Sample of faces and glasses** | | | | | | | |
|  | Latencies (s): glasses | 1.4 | 1.5 | 2.6 | 2.3 | 1.0 | 1.7 |
|  | Latencies (s): faces | 1.8 | 2.8 | 4.0 | 4.1 | 3.2 | 4.2 |
|  | % correct: glasses | 95 | 95 | 100 | 90 | 95 | 95 |
|  | % correct: faces | 86 | 95 | 100 | 90 | 90 | 85 |
|  | | | | | | | |
| **Configural and Featural Processing (d’)** | | | | | | | |
|  | faces blurred | 1.6 | 0.7 | 0.7 | -0.1 | 0.1 | 0.7 |
|  | faces scrambled | 3.8 | 2.3 | 2.9 | 0.9 | 1.9 | 1.9 |
|  | houses blurred | 3.0 | 4.3 | 4.0 | 4.3 | 3.7 | 2.3 |
|  | houses scrambled | 4.9 | 4.6 | 4.6 | 2.9 | 2.2 | 2.3 |
|  | sugar bowls blurred | 4.4 | 4.6 | 6.0 | 4.6 | 6.0 | 2.9 |
|  | sugar bowls scrambled | 4.5 | 2.9 | 6.0 | 2.1 | 6.0 | 6.0 |

Data from controls as well as GH, MH XG from all tests except “configural and featural processing” are taken from (8). LO and BT were tested additionally. Data in the task “configural and featural processing” are taken from (38).

* GH did not participate in this task. For details on the tasks see (8) and (38).
